# Impacts of Wearable Resistance Placement on Running Efficiency Assessed by Wearable Sensors: A Pilot Study

**DOI:** 10.3390/s24134399

**Published:** 2024-07-07

**Authors:** Arunee Promsri, Siriyakorn Deedphimai, Petradda Promthep, Chonthicha Champamuang

**Affiliations:** 1 Department of Physical Therapy, School of Allied Health Sciences, University of Phayao, Phayao 56000, Thailand; 63130474@up.ac.th (S.D.); 63130340@up.ac.th (P.P.); 63130104@up.ac.th (C.C.); 2 Department of Sport Science, University of Innsbruck, A-6020 Innsbruck, Austria

**Keywords:** wearable resistance training, weight vest, running performance, running gait, RunScribe™

## Abstract

Wearable resistance training is widely applied to enhance running performance, but how different placements of wearable resistance across various body parts influence running efficiency remains unclear. This study aimed to explore the impacts of wearable resistance placement on running efficiency by comparing five running conditions: no load, and an additional 10% load of individual body mass on the trunk, forearms, lower legs, and a combination of these areas. Running efficiency was assessed through biomechanical (spatiotemporal, kinematic, and kinetic) variables using acceleration-based wearable sensors placed on the shoes of 15 recreational male runners (20.3 ± 1.23 years) during treadmill running in a randomized order. The main findings indicate distinct effects of different load distributions on specific spatiotemporal variables (contact time, flight time, and flight ratio, *p* ≤ 0.001) and kinematic variables (footstrike type, *p* < 0.001). Specifically, adding loads to the lower legs produces effects similar to running with no load: shorter contact time, longer flight time, and a higher flight ratio compared to other load conditions. Moreover, lower leg loads result in a forefoot strike, unlike the midfoot strike seen in other conditions. These findings suggest that lower leg loads enhance running efficiency more than loads on other parts of the body.

## 1. Introduction

Running is a fundamental form of exercise, physical activity, and sport, with ongoing efforts to develop innovative training methods aimed at improving running performance. One such method gaining prominence is wearable resistance training, which involves strategically attaching additional weight or resistance, such as weighted vests or specialized cuffs, to various parts of the body [1]. Unlike traditional gym-based workouts, wearable resistance training enables athletes to perform sport-specific exercises with added weight, potentially leading to a better transfer of improvements to actual performance [2]. This approach has been extensively used to enhance athletes’ muscular strength, endurance, and overall performance during warm-up routines [3], running [2,4,5], and activities like netball that require a change in direction [6], as an integral component of regular training programs [7]. 

Research has extensively explored the potential benefits of wearable resistance training, particularly its impact on running efficiency, biomechanics, and performance [2,5,8,9,10]. Previous studies reported that runners utilize various load-bearing strategies, e.g., weighted vests [4,11] or cuffs [5], to distribute the loads, potentially enhancing force generation [4] or increasing muscular activity depending on the placement [9,12]. External loading attached directly to the trunk or limbs is thought to provide a vertical load, possibly increasing braking forces and overloading the stretch-shortening cycle [13]. Wearable resistance has also been reported to increase force production, improve sprint performance [2,8], modify stride length and frequency during loaded sprinting, and increase contact time and ground reaction forces [2]. Significant kinematic changes have been observed with different placements of wearable resistance, such as on the calves versus the thighs [2]. Understanding how various placements affect running biomechanics reflecting neuromuscular control [9] is crucial for integrating wearable resistance into training and rehabilitation programs. A recent systematic review comparing wearable resistance and weighted vests for sprint performance found distinct differences in their impact on kinematics [14]. Acute studies indicated that wearable resistance reduced step frequency while weighted vests reduced step length, both increasing sprint times and ground contact times. Long-term adaptations favored wearable resistance for improving sprint times, suggesting varying benefits between equipment types and durations [14]. Despite extensive research on wearable resistance training effects on biomechanics and performance, gaps remain regarding how different placements of wearable resistance across various body parts (e.g., forearms, lower legs, trunk, and combined segments) influence running efficiency. Addressing these gaps could inform more targeted training and rehabilitation programs tailored to optimize running efficiency across diverse popula-tions and performance levels.

The interaction of muscle actions and external forces, e.g., friction, air resistance, ground reaction forces, and gravity, collectively modulate the body’s acceleration during movements [15,16,17]. For running, this underscores the neuromuscular control ability to control motion [17,18], which can be indirectly assessed by focusing on running biomechanics [19]. Recent advancements in wearable sensor technology have enabled continuous monitoring and analysis of running efficiency through various biomechanical variables in different environments used by clinicians, researchers, and athletes [20,21,22]. Acceleration-based wearable sensor systems, incorporating a triaxial accelerometer and gyroscope affixed to the runner’s shoe, have gained considerable recognition for measuring running efficiency variables related to gait dynamics [23,24,25,26,27,28,29]. Systems like RunScribe™ used by several studies [23,24,30,31,32,33,34] provide detailed data on spatiotemporal metrics (step rate, length, contact time, flight time), kinematics (footstrike type, pronation), and kinetics (braking G-forces), offering insights into rhythm, timing, foot motion, and running form [35]. Previous research has shown robust correlations (*r* > 0.9) for these measures and moderate to strong correlations (*r* = 0.4–0.8) for kinematic measures compared with gold standard methods [36,37]. High agreement with standard accelerometry measurement systems has been reported for pronation excursion and pronation velocity, with ICC values ranging from 0.5 to 0.6 [38]. Kinetic variables, e.g., braking G-forces, reflect the forces exerted on the body during each stride, demonstrating good concurrent validity with ICC values ranging from 0.8 to 0.9 [39]. Overall, spatiotemporal metrics, kinematics, and kinetics variables derived from this device have acceptable reliability [40,41,42] and validity [37,43] compared to traditional gold standard devices. 

In addition to advancements in running efficiency analysis, exploring the physiological effects of running with added load is paramount for comprehensive understanding. Basic physiological measures, such as heart rate, blood pressure, and oxygen saturation, offer insights into the cardiovascular and metabolic demands imposed by running with added resistance [44]. Monitoring these physiological responses helps evaluate the body’s adaptation to the increased workload and guides the optimization of training protocols tailored to individual athletes’ needs and goals [45]. Moreover, monitoring perceived exertion can provide valuable information for training since it is a recognized marker of intensity and homeostatic disturbance during exercise [46,47]. Examining the biomechanical and physiological aspects provides a holistic understanding of wearable resistance effects on running performance, maximizing athlete potential while minimizing injury risks or overexertion.

In summary, the current study aimed to explore the effects of applying external loads on running efficiency assessed by acceleration-based wearable sensors. Additionally, altered physiological responses according to running with and without loads were also measured. We hypothesized that variations in running efficiency (spatiotemporal, kinematic, and kinetic variables) and physiological responses (heart rate, respiratory rate, blood pressure, blood oxygen saturation, and perceived exertion) would be evident between running without any load and running with added loads distributed across different body parts. The findings may provide valuable insights for integrating wearable resistance into training routines, empowering individuals to enhance performance effectively while minimizing potential drawbacks.

## 2. Materials and Methods

### 2.1. Participants 

Fifteen regular, recreational male runners with treadmill running experience were recruited for the study. Each participant confirmed their absence of musculoskeletal or neurological issues within the past six months, did not have medical conditions, e.g., hypertension or cardiovascular problems, and had no prior use of wearable resistance equipment during training. Eligible participants underwent a health screening and received essential information from researchers on appropriate attire and footwear, ensuring adequate rest (6–8 h), consumption of a meal 2–3 h before testing, abstaining from consuming energy or alcoholic drinks, and refraining from vigorous activity for at least 24 h.

The sample size was determined through a priori power analysis using G*Power software version 3.1.9.7 (Heinrich-Heine-Universität Düsseldorf, Düsseldorf, Germany), based on findings from a preliminary study that assessed the effects of different wearable resistance placements using acceleration data. The preliminary study indicated an average effect size of 0.42 [9], with α = 0.05 and a desired power of 0.95, suggesting a minimum sample size of *N* = 14. Fifteen young, regular recreational runners volunteered, slightly exceeding the calculated requirement. Male recreational runners were specifically chosen due to the known performance differences between sexes (i.e., sex gap) in recreational settings [48,49], attributed to biological disparities, (e.g., skeletal muscle mass, hormonal factors, and oxidative capacities), which have been accepted as the primary cause [50,51]. Moreover, the diversity in race among amateur runners introduces potential limitations, as outcomes may vary based on training backgrounds and experience levels [52]. This study is, therefore, regarded as an initial exploration due to its emphasis on young male recreational runners. Ethical guidelines outlined in the Declaration of Helsinki were followed, and approval was obtained from the Institutional Review Board of the University of Phayao, Thailand (HREC-UP-HSST 1.3/038/66, Approval Date: 20 August 2023). Written informed consent was obtained from all participants before their involvement. Table 1 summarizes participants’ demographic characteristics and baseline physiological data collected prior to the experiments.

### 2.2. Experimental Procedure

**Figure 1:**
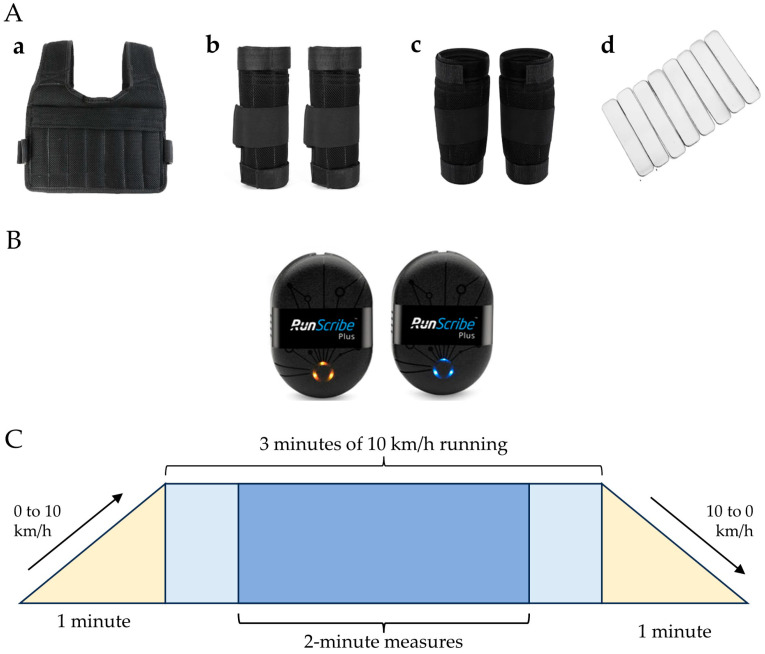
Illustration of (**A**) wearable equipment utilized in the study ((**a**) weighted vest, (**b**) forearm cuffs, (**c**) lower leg cuffs, and (**d**) detachable metal plates); (**B**) RunScribe™ wearable sensors; and (**C**) the experimental protocol.

Each participant began with a warm-up regimen, starting with a 5 min brisk walk (5.5 km/h) on a treadmill (Brightway TT-X10, Shandong Brightway Fitness Equipment Co., Ltd., Jinan, China), followed by a 5-min whole-body stretching routine [10]. Afterward, a 5-min rest period preceded the start of the initial experiment. Participants completed five treadmill running trials in a randomized sequence: one trial without additional load and four trials with a 10% body weight. This load was achieved by inserting detachable metal plates (Figure 1A) into cuffs and vests placed around the forearm, lower leg, and trunk areas, respectively. The selection of a 10% body weight load was based on its documented effectiveness in enhancing running performance [1], while minimizing the risk of injury or overexertion compared to heavier loads and enhancing power output without significantly altering movement mechanics [5]. For the forearm, lower leg, and combined segment conditions, the load was evenly distributed to ensure equal weight allocation to each segment. Metal plates were inserted into the sockets of the cuffs, covering the circumference of the forearms and lower legs. Symmetrical weight distribution was maintained on both the front and back sides of the vest, ensuring the load was evenly distributed across the front and back of the trunk.

Figure 1B depicts the experimental procedure, where treadmill velocity progressively increased from 0 to 10 km/h over one minute, remained constant for 3 min, and then gradually decreased from 10 to 0 km/h within another minute for each running scenario. The chosen speed of 10 km/h aligns closely with the preferred pace of recreational runners [53]. Moreover, before commencing each running trial and after every run, participants were granted a 5-min break, during which they assessed their readiness; if this interval was insufficient, they could extend their rest. Any abnormal symptoms experienced by participants during testing, e.g., dizziness, nausea, vomiting, pain, feelings of insecurity, accidents, or simply a desire to withdraw from participation, constituted grounds for their withdrawal from the study.

### 2.3. Measuring Running Efficiency

A pair of acceleration-based wearable sensors, the RunScribe™ system (Figure 1B), was placed on the shoelaces. These devices provide raw acceleration data to be processed on-board through the proprietary RunScribe™ software version 3.4.0 (470) (Scribe Labs Inc., San Francisco, CA, USA) to derive specific biomechanical variables through the manufacturer’s online dashboard, facilitating the acquisition of three types of biomechanical outcome measures (spatiotemporal, kinematic, and kinetic measures) for analysis [35]. All biomechanical measures were computed for the middle 2-min run of each condition to omit the accelerate and decelerate phases of the running (Figure 1C). 

Spatiotemporal variables encompass footstrike type, pronation excursion, and maximum pronation velocity [35]. Briefly, step rate denotes the frequency of steps taken per minute by a runner. Step length, conversely, measures the linear distance between the heel of one foot and the heel of the same foot in the subsequent step. Contact time represents the duration from the initiation of the heel strike until the conclusion of the toe-off within the same step. Flight time indicates the duration of a running stride when both feet are off the ground and the body is airborne. The flight ratio quantifies the proportion of time during a gait cycle when both feet are off the ground, calculated by dividing the duration of the flight phase by the total duration of the gait cycle. A higher flight ratio is frequently associated with a more efficient running or walking style, as noted by RunScribe™, which attributes this to a combination of shorter contact time, longer flight time, and a higher step rate [35]. When compared to the gold standard technique, prior studies have shown moderate to strong correlations (*r* = 0.4–0.8) for kinematic measurements and robust correlations (*r* > 0.9) for spatiotemporal measures [36,37].

Kinematic variables encompass parameters, e.g., footstrike type, pronation excur-sion, and maximum pronation velocity [35]. In essence, RunScribe™ assigns numerical values to foot strikes, with values ranging from 0 to 6 indicating a heel or rear foot strike, 6 to 10 representing a midfoot strike, and 10 to 16 denoting a forefoot strike. Pronation excursion refers to the total angular movement range between the initial foot strike and the point of maximal pronation, serving as a measure of foot roll, a typical pronation metric [25]. RunScribe™ provides two figures for pronation: from foot strike to maximum pronation (−2 to −20 degrees) and from maximum pronation to toe-off (−10 to 15 degrees). Negative values signify pronation, while positive values indicate supination or outward rolling. Furthermore, maximum pronation velocity refers to the peak angular velocity at which the foot pronates between the initial foot strike and maximal pronation, signifying the speed of foot pronation in degrees per second [25]. RunScribe™ reports a range from 200 to over 1000 degrees per second. It has been reported that there is high agreement with standard accelerometry measurement systems for pronation excursion and pronation velocity, with ICC values ranging from 0.5 to 0.6 [38].

Kinetic variables encompass impact Gs and braking Gs [35]. Impact Gs represents the vertical component of peak Gs, which correlates with the ground impact force experienced at foot strike. According to RunScribe™, braking Gs typically range from 4 to 13 Gs, with lower values considered more favorable. Braking Gs denotes the horizontal component of Peak Gs, indicating the braking forces experienced at foot strike. Additionally, the system’s kinetic measures, especially acceleration data, demonstrated good concurrent validity, with ICC values ranging from 0.8 to 0.9 [39].

### 2.4. Measuring Physiological Responses

At baseline (before experiments) and the end of each run, participants underwent immediate measurements of basic physiological responses, including heart rate, respiratory rate, blood pressure, blood oxygen saturation (SpO2), and the Borg Rating of Perceived Exertion (RPE) Scale. Blood pressure and heart rate were recorded using an upper arm blood pressure monitoring machine (Omron 5 Series Wireless Upper Arm Blood Pressure Monitor, Omron Corporation, Kyoto, Japan). Blood pressure is the pressure exerted by circulating blood against the walls of blood vessels, comprising two primary measurements: systolic blood pressure, which represents the pressure exerted on the vessel walls when the heart contracts and pumps blood, and diastolic blood pressure, which indicates the pressure when the heart is in a relaxed state between beats [44]. Heart rate refers to the number of times the heart beats per minute, typically measured as an indicator of cardiovascular health and exertion during physical activity [44]. Blood oxygen saturation was assessed using a pulse oximeter with an alarm (P300 Intelli IT HPO-300T, Omron Corporation, Kyoto, Japan). SpO_2_ refers to the percentage of oxygen bound hemoglobin relative to the total hemoglobin in the blood, reflecting a measure of how effectively oxygen is being carried from the lungs to the body’s tissues [44]. Respiratory rate is the number of breaths a person takes per minute, which is an important indicator of respiratory health and function. was manually measured by placing a hand on the chest to feel the rise and fall with each breath [44]. The Borg’s rating of perceived exertion (RPE) is a subjective measure utilized to gauge an individual’s perception of effort during physical activity, offering a numerical rating that reflects their perceived level of exertion [54,55]. The RPE scale ranges from 6 to 20, with corresponding verbal anchors to assist individuals in interpreting the ratings, with 6 representing no exertion at all (rest) and 20 indicating maximum exertion [54,55]. 

### 2.5. Statistical Analysis

The statistical analyses were conducted using SPSS software version 26.0 (IBM SPSS Statistics, SPSS Inc., Chicago, IL, USA). A significance level was set at α = 0.05. The normal distribution of the variables under consideration was assessed using the Shapiro–Wilk test. A one-way repeated-measures ANOVA was employed to examine the impact of different running loads on running efficiency (spatiotemporal, kinematic, and kinetic variables) and physiological responses (blood pressure, heart rate, respiratory rate, and blood oxygen saturation; Borg Rating of Perceived Exertion). Effect sizes (Partial Eta Square; η_p_^2^) and observed power (1 − β) were also documented. Post hoc analyses were conducted with the alpha level set to α < 0.005 to manage the familywise error rate across the five running conditions, adjusting for multiple comparisons using Bonferroni correction [55].

## 3. Results

### 3.1. Running Efficiency

All participants completed the running tests without experiencing any discomfort and did not meet the withdrawal criteria. Table 2 shows the main findings regarding the impact of wearable resistance placements observed on specific running biomechanical variables. These variables include contact time (F_(2.18,30.46)_ = 16.89, *p* < 0.001, η_p_^2^ = 0.547, 1 − β = 1), flight time (F_(2.65,37.11)_ = 6.77, *p* = 0.001, η_p_^2^ = 0.326, 1 − β = 0.956), flight ratio (F_(2.58,36.15)_ = 15.72, *p* < 0.001, η_p_^2^ = 0.529, 1 − β = 1), footstrike type (F_(2.28,31.91)_ = 16.54, *p* < 0.001, η_p_^2^ = 0.542, 1 − β = 1), and braking Gs (F_(2.31,32.41)_ = 7.99, *p* = 0.003, η_p_^2^ = 0.311, 1 − β = 0.901).

**Figure 2:**
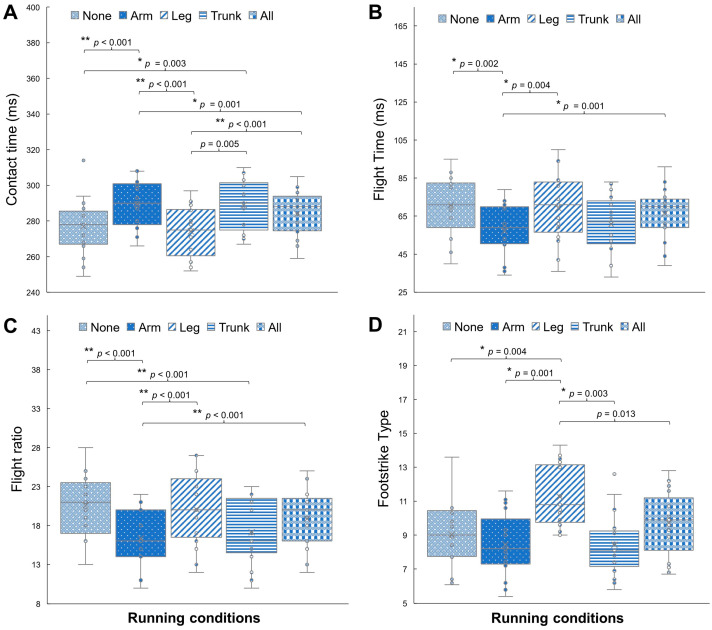
Post hoc comparisons of (**A**) contact time, (**B**) flight time, (**C**) flight ratio, and (**D**) footstrike type between five running conditions: running with no load (None) and running with added loads on the forearms (Arm), lower legs (Leg), trunk (Trunk), and combined segments (All). Significant differences are indicated as * *p* < 0.005 and ** *p* < 0.001.

In Figure 2, post hoc tests reveal significant differences in specific pairs of running conditions. For contact time (Figure 2A), running without additional load showed a shorter contact time compared to running with added loads on the forearms (*p* < 0.001) and trunk (*p* = 0.003). Additionally, running with added loads on the forearms displayed a shorter contact time than running with added loads on combined segments (*p* < 0.001). Running with added loads on the lower legs exhibited a shorter contact time than running with added loads on the forearms (*p* = 0.001), trunk (*p* = 0.005), and combined segments (*p* < 0.001).

Regarding flight time (Figure 2B), running with added loads on the forearms displayed a shorter flight time compared to running without any load (*p* = 0.002), running with added loads on the lower legs (*p* = 0.004), and running with added loads on combined segments (*p* = 0.001). Additionally, for flight ratio (Figure 2C), running with added loads on the lower legs resulted in a higher flight ratio than running without any load (*p* = 0.001) and running with added loads on the forearms (*p* < 0.001). Furthermore, running with added loads on the forearms exhibited a higher flight ratio than running with added loads on the trunk (*p* = 0.001).

In terms of footstrike type (Figure 2D), running with added loads on the lower legs displayed a different footstrike type compared to running without any load (*p* = 0.004), running with added loads on the forearms (*p* = 0.001), and running with added loads on the trunk (*p* = 0.003). Running with added loads on the lower legs exhibited a forefoot strike, whereas the other running conditions showed a midfoot strike. Lastly, regarding braking Gs, although the main effects indicated a significant difference for the within-subject effect, running with added loads on the lower legs tended toward higher braking Gs compared to running with added loads on the forearms (*p* = 0.006).

### 3.2. Physiological Responses

Table 3 illustrates the main effect of running load, as evidenced by systolic blood pressure (F_(2.29,32.17)_ = 10.61, *p* < 0.001, η_p_^2^ = 0.431, 1 − β = 0.993). Post hoc tests revealed that systolic blood pressure significantly increased immediately after runs with added loads on the lower legs (Leg) compared to running without any load (None, *p* = 0.004) and running with added loads on the forearms (Arm, *p* = 0.006), trunk (Trunk, *p* = 0.002), and combined segments (All, *p* ≤ 0.001). However, participants consistently reported a perceived exertion level ranging from 10 to 11 across all running conditions with added load, indicating a feeling of fairly light perceived exertion.

## 4. Discussion

The present study explored the impact of added loads to—the forearms, lower legs, trunk, combinations of segments (forearms, lower legs, and trunk), and no load—on running efficiency assessed by wearable sensors and physiological responses. The findings reveal that load distribution influences specific spatiotemporal variables (contact time, flight time, and flight ratio) and one kinematic variable (footstrike type). Specifically, adding external loads to the lower legs mirrors running without additional weight, resulting in shorter contact time, longer flight time, and a higher flight ratio. However, running with added loads on the lower legs prompts a different footstrike type characterized by a forefoot strike, in contrast to the midfoot strike observed in other running conditions. Regarding physiological responses, only running with added loads significantly affects systolic blood pressure, with running while loaded on the lower legs exhibiting higher levels compared to running without loads and running with added loads distributed on other body positions. Based on the current findings, two key points can be discussed. 

First, the consistent findings of shorter contact time, longer flight time, and a higher flight ratio during running with added loads on the lower legs can be attributed to the need for runners to generate more power to rapidly lift their legs. This results in a shorter ground contact time and a longer airborne phase as runners compensate for the added resistance [56]. Although the specific mechanisms influencing running gait are not fully understood, it is assumed that running with added loads directly placed on the lower legs challenges the sensorimotor system, particularly through increased muscular output [57] and improved muscular coordination [58] in the lower limb muscles responsible for locomotion. Additionally, the shift from midfoot to forefoot strike due to weight distribution changes raises questions about long-term injury risks. Forefoot strikes exhibit lower patellofemoral stress and knee frontal plane moments than rearfoot strikes, potentially reducing knee injury risk [59,60]. However, forefoot strikes with increased contact forces may increase the probability of ankle and foot injuries due to greater compression of the ankle joint and higher loading on the ankle plantar flexors and Achilles tendon [59]. This alteration in footstrike patterns under added load conditions may change muscle activation dynamics and joint loading, increasing the risk of both acute and chronic injuries. Therefore, it is crucial to consider the long-term impacts of altered footstrike patterns and emphasize tailored training and injury prevention strategies. However, a recent systematic study found no consistent link between specific foot strike types and injury incidence, suggesting that running-related injuries are influenced by multiple factors, including biomechanics, training loads, and individual variability, making it insufficient to predict injury risk based solely on foot strike type [61]. 

Second, regarding physiological measures, running with added loads specifically on the lower legs shows a particularly pronounced increase in systolic blood pressure compared to other conditions, highlighting the importance of load distribution and its impact on cardiovascular demand during running. Loads on the lower legs may impose a greater cardiovascular workload, potentially due to increased muscle mass and gravitational forces acting on the lower extremities, as observed by the increased intensity of vertical motion [57]. This heightened cardiovascular response reflects the enhanced muscular work required to overcome the added resistance during running, thereby increasing cardiac output to meet the elevated metabolic demands [62]. However, participants showed no significant difference in diastolic blood pressure across the various running conditions. Elevated systolic blood pressure post-exercise can indicate increased cardiac strain, which may have implications for overall cardiovascular health and exercise tolerance [45]. Moreover, the lack of significant changes in diastolic blood pressure across different running conditions suggests that the cardiovascular system can effectively regulate blood flow and maintain arterial pressure within a normal range during exercise, even when subjected to added physical stress [45]. Similar to previously reported findings, dynamic upright exercises like running typically elicit a progressive increase in systolic blood pressure and minimal changes in diastolic blood pressure [45]. Collectively, the increased cardiovascular demand encountered while running with lower leg loads may result in a higher cardiac workload, which could influence an individual’s capacity to maintain extended physical activity and affect their overall cardiovascular fitness. Despite the observed changes in systolic blood pressure, participants reported a perceived exertion level ranging from 10 to 11 across all running conditions with added load, as measured by the Borg Scale [54,55], indicating a subjective perception of fairly light exertion imposed by all running conditions. This alignment between perceived exertion and physiological responses suggests a degree of adaptation or tolerance to the added load, wherein participants perceive the task as less demanding than the physiological stress would suggest.

From a practical perspective, the current findings highlight the relationship between load distribution, spatiotemporal variables, and footstrike patterns in running. These insights reveal how external loads, particularly on the lower legs, influence runners’ biomechanical adjustments and physiological responses for performance optimization. Based on these findings, several implications can be acknowledged. First, running with added loads on the lower legs significantly increases systolic blood pressure compared to other conditions, suggesting a greater cardiovascular workload that may enhance cardiovascular fitness and endurance. However, it’s crucial to note that adding loads to the lower legs increases impact forces with each stride, raising the risk of overuse injuries. Therefore, appropriate footwear with shock absorption during forefoot running is imperative [63]. Second, the use of resistance loads on the lower legs can modify a runner’s kinematics by improving mediolateral stability during running but increasing vertical movement intensity [57], which requires careful consideration of footwear [63]. The application of resistance loads should be approached cautiously and progressively to prevent overtraining or injury [64]. Third, coaches can implement progressive training protocols incorporating wearable resistance to target biomechanical adjustments beneficial for enhancing performance. Adjusting the placement and amount of resistance can influence stability and movement intensity during running, thereby improving efficiency [57]. Runners and coaches should carefully monitor training loads and ensure gradual progression to prevent injury. Fourth, the study’s findings suggest potential implications for the design of wearable resistance equipment, particularly cuffs and metal plates applied to the forearms and lower legs. Future developments could focus on designing these devices to cover the length of these segments while ensuring they do not restrict joint movement or cause irritation, such as around the popliteal fossa. Incorporating soft garment materials alongside these devices is recommended to enhance comfort and minimize skin irritation. Balancing the need for added resistance with ergonomic considerations will be crucial to optimizing the effectiveness of wearable resistance training in improving running biomechanics and performance. 

Overall, it is recommended that coaches and runners utilize acceleration-based wearable sensors due to their user-friendly nature and their effectiveness in monitoring running efficiency. These sensors are suitable for real-world settings and do not require laboratory-based equipment. Additionally, measuring basic physiological responses is a standard method for assessing cardiovascular fitness. This can be carried out anywhere by anyone without the need for specialized devices. Such accessibility enhances the ability to monitor runner performance, thereby facilitating more informed training adjustments and performance evaluations.

## 5. Limitations

The current study is subject to several notable concerns. First, the short duration of participant running—only 5 min with 3 min at a stable speed—may limit the capture of potential variations or effects, and extending the running period could reveal differences in results. Second, instead of running at a percentage of their self-preferred maximum speed, each participant ran at a set pace, which may have ignored individual differences in running ability and intensity. A more dynamic and individualized approach might be provided by using a methodology that considers a proportion of the participants’ maximal speed. Third, the current study focused on recreational male runners who were amateur participants, which may affect running performance, as previously reported [65]. This demographic specificity may limit the generalizability of the findings to broader populations, such as female runners or individuals of varying skill levels. While the research acknowledges the importance of discussing potential biomechanical responses and running efficiency among diverse demographics, including females [53,66,67] and varying skill levels [52], the specific participant demographics could affect the applicability of results across different groups. Fourth, the absence of VO2 max measurements limits understanding of cardiovascular fitness and aerobic endurance, despite focusing on heart rate, respiratory rate, blood pressure, and SpO_2_. Equipment constraints precluded direct VO2 max assessment, hindering a comprehensive exploration of physiological responses. Fifth, sole reliance on perceived exertion (RPE) for assessing exertion levels restricts the study’s depth. Integrating measures like heart rate variability (HRV) would offer a more comprehensive view of physiological responses, though equipment limitations prevented HRV inclusion [68]. Sixth, since the current study focuses on using acceleration-based wearable sensors to measure spatiotemporal, kinematics, and kinetics variables, further study of other kinematic variables (ankle, knee, hip joint angles) is of interest [69]. Seventh, although our study ensured that all participants completed the running tests without experiencing any discomfort and did not meet the withdrawal criteria, implementing a gradual load introduction could help participants adapt to the new conditions, thereby minimizing risks. Moreover, the disparities between treadmill and overground running biomechanics [70] underscore the importance of cautious translation of these findings to track settings. The current study’s reliance on treadmill running, chosen to standardize biomechanical variables and control environmental factors, may not fully replicate the conditions of overground running [70]. Treadmill running lacks the variability of outdoor terrain, e.g., inclines, declines, and uneven surfaces, which can significantly influence running biomechanics and performance outcomes. Factors like wind resistance and environmental temperature variations are also absent in treadmill settings but can affect physiological responses during overground running. Eighth, the current study’s limitation lies in its observational design. To fully understand the lasting impacts and ideal placement of wearable resistance on running efficiency and biomechanics, long-term investigations are essential. Previous systematic reviews underscored contrasting outcomes between short-term and prolonged studies: Short-term findings suggested wearable resistance lowered step frequency and weighted vests decreased step length, resulting in longer sprint times and ground contact periods [14]. Conversely, extended use favored wearable resistance for enhancing sprint times, implying diverse benefits across equipment types and durations [14]. 

Future research should consider expanding participant diversity to further explore these nuances and enhance the broader relevance and impact of findings in running biomechanics and performance optimization. While our meticulous documentation of sensor placement and data collection ensures reproducibility, the findings may not fully generalize to overground settings where these natural elements play a critical role in shaping running mechanics and physiological adaptations. Future research should aim to validate these findings in diverse overground conditions to enhance ecological validity and broaden the practical applications of our results. 

## 6. Conclusions

Investigating the effects of load distribution on running efficiency using wearable sensors and physiological responses reveals that adding loads to the lower legs produces effects similar to running without added weight, namely shorter contact time, longer flight time, and a higher flight ratio. However, running with added loads on the lower legs results in a forefoot strike type, unlike other conditions. Physiologically, running with added loads on the lower legs significantly increases systolic blood pressure post-exercise, indicating a greater cardiovascular workload. Despite this, participants reported a consistent perception of exertion around 11 on the Borg RPE scale across all conditions, suggesting a subjective perception of fairly light exertion. Overall, these findings suggest that loading the lower legs enhances specific aspects of running efficiency, offering valuable insights for optimizing performance while considering physiological demands and perceived exertion.

## Figures and Tables

**Table 1:** Characteristics of participants (mean ± SD).

	Mean	Range
Age (years)	20.3 ± 1.0	22.0–19.0
Mass (kg)	64.7 ± 6.5	77.0–53.0
Height (cm)	170.3 ± 5.6	180.0–163.0
Body mass index (kg/m^2^)	22.3 ± 2.1	26.6–19.2
Weekly mileage (km)	20.1 ± 11.1	40.0–5.0
Heart rate (bpm)	72.1 ± 13.1	52–97
Respiratory rate (bpm)	16.1 ± 1.9	12–19
Systolic blood pressure (mmHg)	131.5 ± 10.7	114–141
Dyastolic blood pressure (mmHg)	76.1 ± 9.0	65–96
Blood oxygen saturation (SpO2 (%))	97.3 ± 0.9	96–99
Borg Rating of Perceived Exertion (RPE) scale	6.6 ± 1.5	6–8

**Table 2:** Main effects of wearable resistance placements on the running efficiency of treadmill running with no load (None) and running with added loads on the forearms, lower legs, trunk, and combination of these segments (All) (mean ± SD, * *p* ≤ 0.001).

Variable	None	Forearms	Lower Legs	Trunk	All	*p*-Value
Step rate (steps/min)	173 ± 6.6	173 ± 6.8	175 ± 6.8	172 ± 6.7	172 ± 6.1	0.076
Step length (m)	0.91 ± 0.05	0.91 ± 0.05	0.91 ± 0.05	0.91 ± 0.05	0.91 ± 0.05	0.102
Contact time (ms)	277 ± 16.8	289 ± 13.8	274 ± 14.6	289 ± 14.6	283 ± 13.3	0.000 *
Flight time (ms)	70.6 ± 16.4	57.2 ± 14.2	69.3 ± 18.7	60.8 ± 15.5	66.3 ± 14.3	0.001 *
Flight ratio	20.6 ± 4.2	16.3 ± 3.9	20.1 ± 4.9	17.1 ± 4.3	18.9 ± 3.8	0.000 *
Footstrike type	9.0 ± 2.0	8.3 ± 1.9	11.2 ± 1.8	8.4 ± 1.9	9.7 ± 2.0	0.000 *
Pronation excursion (°)	−11.9 ± 3.8	−12.3 ± 4.7	−12.9 ± 6.1	−12.4 ± 4.0	−12.3 ± 4.5	0.458
Maximum pronation velocity (°/s)	571 ± 128	553 ± 138	523 ± 164	549 ± 118	544 ± 138	0.318
Impact Gs (G)	8.4 ± 1.7	8.3 ± 1.6	8.8 ± 1.2	8.5 ± 1.7	8.7 ± 1.8	0.423
Braking Gs (G)	7.4 ± 2.2	6.9 ± 2.0	8.3 ± 2.1	7.0 ± 1.7	7.3 ± 1.9	0.003

**Table 3:** The main effects of wearable resistance placements on physiological responses of treadmill running, comparing conditions with no additional load (None) and those with loads applied to the forearms, lower legs, trunk, and combination of these segments (All) (mean ± SD, * *p* ≤ 0.001).

Variable	None	Forearms	Lower Legs	Trunk	All	*p*-Value
Heart rate (bpm)	109 ± 18	110 ± 21	115 ± 22	110 ± 19	110 ± 19	0.070
Respiratory rate (bpm)	24.5 ± 4.1	24.5 ± 3.5	25.8 ± 3.7	25.1 ± 4.0	26.6 ± 3.5	0.112
Systolic blood pressure (mmHg)	141 ± 10 ^a^	144 ± 14 ^b^	163 ± 17	138 ± 11 ^c^	143 ± 17 ^d^	<0.001 *
Dyastolic blood pressure (mmHg)	76 ± 10	74 ± 9	86 ± 15	80 ± 11	77 ± 113	0.024
Blood oxygen saturation (SpO_2_ (%))	96.5 ± 1.1	96.9 ± 1.1	97.1 ± 1.5	96.3 ± 1.0	96.2 ± 1.4	0.122
Borg Rating of Perceived Exertion (RPE) scale	11.0 ± 2.8	11.9 ± 2.2	10.6 ± 3.2	11.5 ± 2.5	11.5 ± 2.0	0.140

Note: The post hoc comparisons show significant differences between each pair; ^a^ = None vs. Lower Legs (*p* = 0.004), ^b^ = Forearms vs. Lower Legs (*p* = 0.006), ^c^ = Trunk vs. Lower Legs (*p* = 0.002), and ^d^ = All vs. Lower Legs (*p* ≤ 0.001).

## Data Availability

The data presented in this study are available on request from the corresponding author due to ethical reasons.
